# Artificial intelligence-based radiogenomics reveals the potential immunoregulatory role of COL22A1 in glioma and its induced autoimmune encephalitis

**DOI:** 10.3389/fimmu.2025.1562070

**Published:** 2025-03-06

**Authors:** Bingchao Yan, Qian Chen, Dacheng Wang, Leili Ding, Jingfeng Qu, Renfei Du, Wenjie Shi, Ulf D. Kahlert, Zhengquan Yu

**Affiliations:** ^1^ Department of Neurosurgery, The First Affiliated Hospital of Soochow University, Suzhou, China; ^2^ Department of Neurosurgery, The Affiliated Xuzhou Municipal Hospital of Xuzhou Medical University, Xuzhou, China; ^3^ Department of Neurosurgery, The Second Affiliated Hospital of Guilin Medical University, Guangxi, China; ^4^ Molecular and Experimental Surgery, Clinic for General-, Visceral -, Vascular- and Transplantation Surgery, Medical Faculty and University Hospital Magdeburg, Otto-von-Guericke University, Magdeburg, Germany; ^5^ Nantong University, Nantong, China; ^6^ Chifeng Municipal Hospital, Chifeng, China

**Keywords:** artificial intelligence, tumor microenvironment, COL22A1, autoimmune encephalitis, radiomics

## Abstract

**Background:**

The tumor microenvironment plays a crucial role in the progression of both glioma and glioma-induced autoimmune encephalitis. However, there remains a significant lack of effective therapeutic targets for these diseases.

**Method:**

We collected 54 CT images of glioma patients and 54 glioma-induced autoimmune encephalitis patients, respectively. Radiomics features were extracted from tumors and encephalitis regions using Python, followed by dimensionality reduction via random forest and lasso regression, and construction of radiomics-based risk scores. Genomic data matched with clinical information were analyzed to identify key prognostic genes significantly associated with risk scores. Gene expression was validated by immunohistochemistry using our clinical samples. Immune infiltration was evaluated using five algorithms (MCP-counter, EPIC, TIMER, QUANT and IPS). The association between hub genes and immune checkpoint markers as well as immunoregulation-related genes was also analyzed using Spearman correlation.

**Results:**

We identified 980 radiomics features both in glioma and encephalitis patient images and selected four key features through lasso regression to build a radiomics-based risk score. COL22A1 was strongly correlated with the risk score and identified as the hub prognostic gene. COL22A1 expression was higher in glioblastoma tissues and cell lines, and correlated with clinical factors such as higher age, WHO grade, and IDH mutation status. Immune infiltration analysis indicated associations with diverse immune and stromal cell populations, including CD8^+^T cells, macrophages, and CAFs. COL22A1 was also positively correlated with immune checkpoints and immune-regulated genes.

**Conclusion:**

Our study highlights the critical role of COL22A1 in gliomas and glioma-Induced Autoimmune Encephalitis, demonstrating its strong association with poor prognosis and its significant involvement in tumor immune regulation.

## Introduction

Glioblastoma (GBM) represents the most common and severe type of malignant primary brain tumor, making up roughly 30% of all primary brain tumors (1). Glioma-Induced Autoimmune Encephalitis is a Common Paraneoplastic Syndrome of GBM. This disease predominantly affects the elderly and shows a higher prevalence in men compared to women, with annual incidence rates ranging between 0.59 and 5 cases per 100,000 individuals (2, 3). However, There remains a significant lack of effective therapeutic targets for these diseases.

Gliomas are classified into four grades based on histopathology, from low-grade (I and II) to high-grade (III and IV, with IV being GBM), reflecting their levels of malignancy (4). Recent advancements have introduced genetic criteria such as TERT promoter mutations, EGFR amplification, and chromosomal changes for a more precise diagnosis and tailored treatment of glioblastoma IDH-wildtype (5). Despite advances in understanding its molecular biology and pathogenesis, GBM remains extremely difficult to manage, with nearly inevitable tumor recurrence. Patients typically have a median survival of approximately 15 months and a five-year survival rate of 6.8%, underscoring the urgent need for new treatment approaches for GBM patients (1, 6). The primary treatment modalities for GBM include surgical resection, chemotherapy, and radiation therapy. Advances in surgical resection, such as frameless stereotaxy and brain mapping, have enhanced the precision of tumor removal, significantly influencing survival rates (7, 8). Standard post-surgical treatments, including temozolomide and radiation therapy, target residual cancer cells, extending progression-free survival but not fully eradicating the disease (6, 9). Improved radiation techniques now more accurately focus on cancer cells while protecting healthy tissue, addressing the challenges of hypoxic tumor environments to enhance treatment efficacy (10). Recent progress in comprehending the molecular and genetic underpinnings of GBM has spurred the creation of novel treatments targeting specific genetic alterations and signaling cascades. For instance, immunotherapy has shown potential as a treatment option, though overall efficacy remains moderate in GBM clinical trials, with certain patients achieving extended survival following therapy (11). The ReACT Phase II trial demonstrates that rindopepimut, combined with bevacizumab, significantly enhances survival and induces a strong immune response against EGFRvIII in patients with relapsed GBM, showing promising results in both progression-free and OS rates (12). In addition, another study reveals that the recombinant poliovirus/rhinovirus chimera PVSRIPO acts as a powerful intratumoral immune adjuvant in GBM, enhancing antitumor immunity by promoting dendritic cell and T cell infiltration and inducing specific cytotoxic T lymphocyte responses against tumor antigens (13). However, GBM employs a variety of mechanisms to evade immune detection and suppression, including an intact blood-brain barrier (BBB) that restricts immune cell entry, a tumor microenvironment (TME) that suppresses immune responses, and the manipulation of immune checkpoints and other key pathways (14–16). As a result, phase III clinical trials involving immune checkpoint inhibitors (ICIs) and vaccine therapies for GBM have yielded disappointing outcomes (17).

Autoimmune encephalitis (AE), characterized by brain inflammation due to an aberrant immune response targeting self-antigens in the central nervous system, represents a diverse group of disorders (18). While infections are the most common triggers, recent scholarly reports suggest that gliomas may also induce autoimmune encephalitis through mechanisms such as altered immune system functions and disrupted immune surveillance (19). The presence of gliomas might lead to an increased production of autoantibodies against brain antigens, potentially triggering this autoimmune condition. This underscores the intricate relationship between tumor-related mechanisms and immune system dysfunction, highlighting the critical need for meticulous clinical evaluation and monitoring in glioma patients. Therefore, elucidating the immune complexities of GBM and identifying its immunosuppressive factors are key to enhancing immunotherapy. Similarly, recognizing what triggers gliomas to cause autoimmune encephalitis is vital for improving diagnostic precision and refining treatment approaches, ultimately minimizing misdiagnosis and delays in therapy.

In this study, we identified COL22A1 as a novel shared biomarker in glioblastoma (GBM) and its induced autoimmune encephalitis (AE) through radiogenomics. Building on this foundation, we further explored the immunological characteristics of this gene, providing valuable insights for the precise diagnosis and treatment of both diseases.

## Materials and methods

### Data obtain and prepared

We collected 54 CT images of glioma patients and 54 Glioma-Induced Autoimmune Encephalitis patients from our hospital. All images were normalized and low-quality images were removed. The genomic information of the patients was obtained by matching clinical information from the corresponding public databases. In addition, clinical tissue samples and baseline data were collected for subsequent validation of core gene expression.

### Radiomics feature extra and related riskscore

We used 3D slice to first label the DICOM-formatted glioma images and Glioma-Induced Autoimmune Encephalitis images. In the next step, we used Python to perform feature extraction for both groups and used Random Forest to perform dimensionality reduction analysis on the common features. Further, we performed further feature selection on the results extracted by Random Forest, where we used lasso regression. Finally, we built Radiomics related Riskscore based on the regression coefficients and feature expressions from lasso regression.

### Identify hub prognosistic gene associated with riskscore

We performed batch survival analysis on the matched genomic data and defined genes as significant for influencing the prognosis of gliomas if HR was greater than 3 and p less than 0.05. Subsequently, we correlated these screened genes with riskscore by Spearman method and identified novel and strongly correlated candidate genes.

### Expression validate and survival analysis of hub gene

We first verified the expression of the gene in tissue samples from public database. Then we download cell line expression data of this gene from The Cancer Cell Line Encyclopedia(CCLE) database, ggplot2 was used for visualization. Subsequently, we collected 20 gliomas as well as paired paraneoplastic tissue samples from the hospital’s brain center and performed immunohistochemical analyses, and the experimental steps were performed in strict accordance with standardized procedures. Further, we performed a correlation analysis with the clinical data based on the differences in the expression of the gene at the protein level in the tumor samples. Finally, we evaluated the effect of the gene on the survival events of the patients, and the outcome indicators were Overall Survival(OS), Disease-Free Survival(DFS), and Progression-Free Survival(PFS), respectively. In addition, we likewise analyzed the correlation of core genes with patient clinical information in public databases for clinical variables including age, gender, race, WHO classification, IDH status, and 1q/19q codeletion.

### Enrichment analysis of hub gene

To explore the potential biological mechanism of this gene, we performed single-gene GSEA analysis. We first categorized the samples into high and low groups by the median expression value, followed by single-gene GSEA analysis using the R package cluster, where an absolute NES value greater than 1 was considered a meaningful enrichment result. Subsequently, we used the ggplot2 package to visualize the enrichment results of interest.

### Immune infiltration analysis of hub gene

To explore the relationship between the hub gene and immune cell infiltration within the tumor microenvironment, we conducted a comprehensive immune infiltration analysis using five distinct algorithms, they are Microenvironment Cell Populations counter(MCP-counter), Estimating the Proportions of Immune and Cancer cells(EPIC), Tumor Immune Estimation Resource(TIMER), Quantify the immune cell types(QUANT), and Immunophenoscore(IPS). These algorithms were selected due to their complementary strengths in quantifying immune cell proportions and activity across diverse datasets. Gene expression data for the hub gene were extracted from TCGA datasets, and normalized as appropriate for each algorithm. Statistical analyses were conducted to assess the correlation between hub gene expression and immune infiltration levels, using Spearman correlation. These analyses provided a multi-faceted view of immune infiltration and its association with the hub gene, offering insights into its potential role in immune regulation within the tumor microenvironment.

### Immune check points and immune regulate factor with hub genes

To investigate the association between the hub gene and immune checkpoint molecules, we analyzed the correlation between hub gene expression and key immune checkpoint markers, including BTLA, CD274, CD96, PDCD1LG2, ICOS, IDO1, CD86, LAG3, and PDCD1. Gene expression data were obtained from TCGA public database, and normalized prior to analysis. Spearman correlation analyses were conducted to determine the strength and direction of the association between the hub gene and immune checkpoint molecules. Additionally, we visualized these relationships using scatter plots, generated in ggplot2, to better understand the interaction between the hub gene and immune checkpoints.

## Results

### Radiomics selection and risksocre build

**Figure 1:**
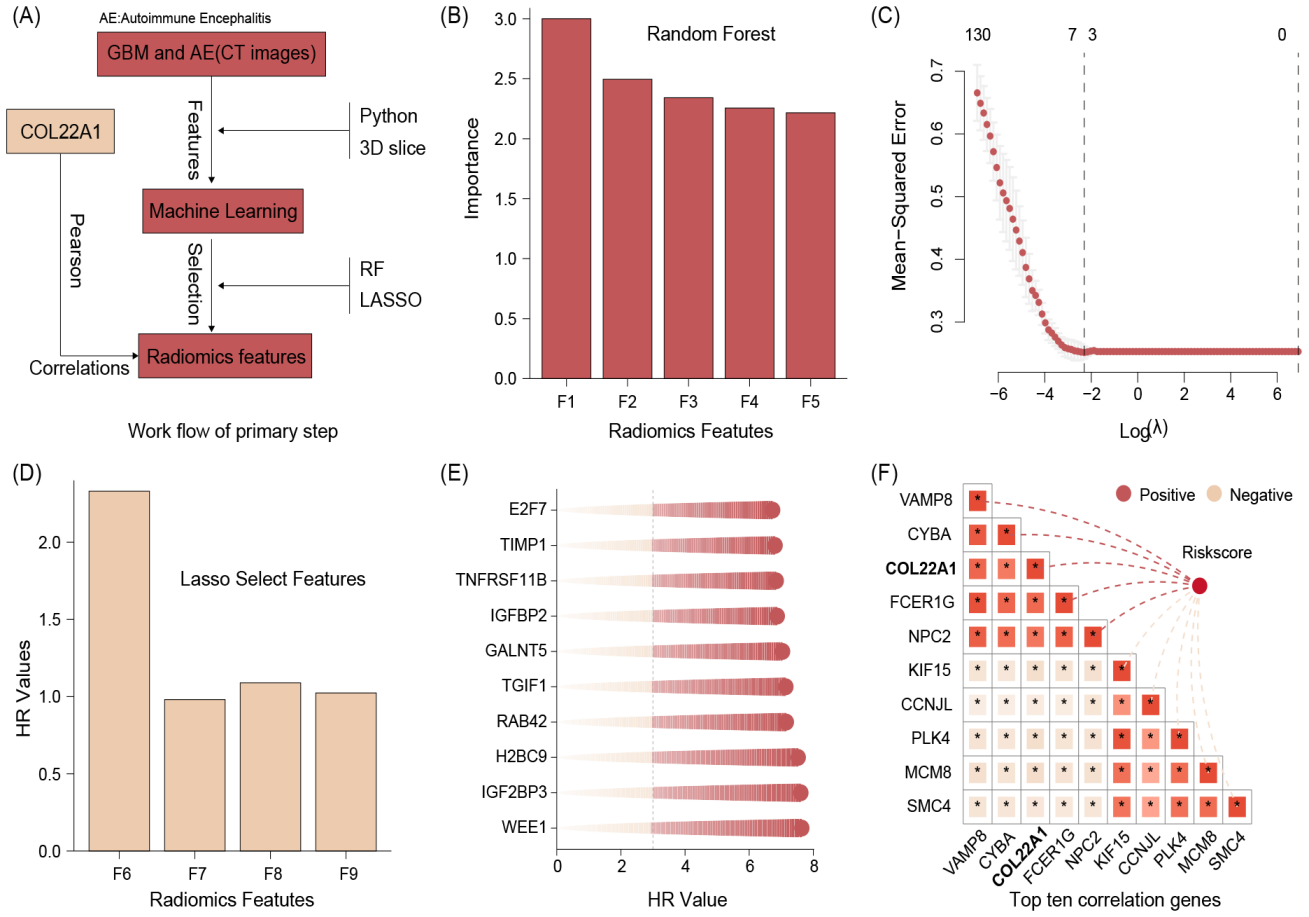
The identification process of core genes **(A)**,The top 5 importance radiomics features **(B)**. Lasso regression results suggested that a total of 4 Radiomics features were selected **(C, D)**. We then performed risk score calculation based on the regression coefficients and feature expression. The top 10 glioma prognostic genes **(E)**. The correlation between prognostic genes and riskscore, and the results suggested that the top 10 genes were VAMP8, CYBA, COL22A1, FCER1G, NPC2, KIF15, CCNJL, PLK4, MCM8, and SMC4 **(F)**.

The identification process of core genes is shown in **Figure 1A**. From all patients image, we collect 980 radiomics features and after conduct random forest a total of 131 radiomics features were selected. We show the top 5 importance radiomics features in **Figure 1B**. Further, our study found that lasso regression analysis of the above results suggested that a total of 4 Radiomics features were selected (**Figures 1C, D**). We then performed risk score calculation based on the regression coefficients and feature expression.

### COL22A1 was positive with radiomics related risksocre

On the other hand, we screened a total of 2113 glioma prognostic genes with HR greater than 3 and p less than 0.05 by batch survival analysis and showed the top 10 gene names in **Figure 1E**. Finally, we performed correlation analysis between the above genes and riskscore, and the results suggested that the top 10 genes were VAMP8, CYBA, COL22A1, FCER1G, NPC2, KIF15, CCNJL, PLK4, MCM8, and SMC4 (**Figure 1F**). Synthesizing the results of the literature review and correlation analysis, we finally identified COL22A1 as a follow-up study.

### COL22A1 was high expression in GBM and negative with survival rate

**Figure 2:**
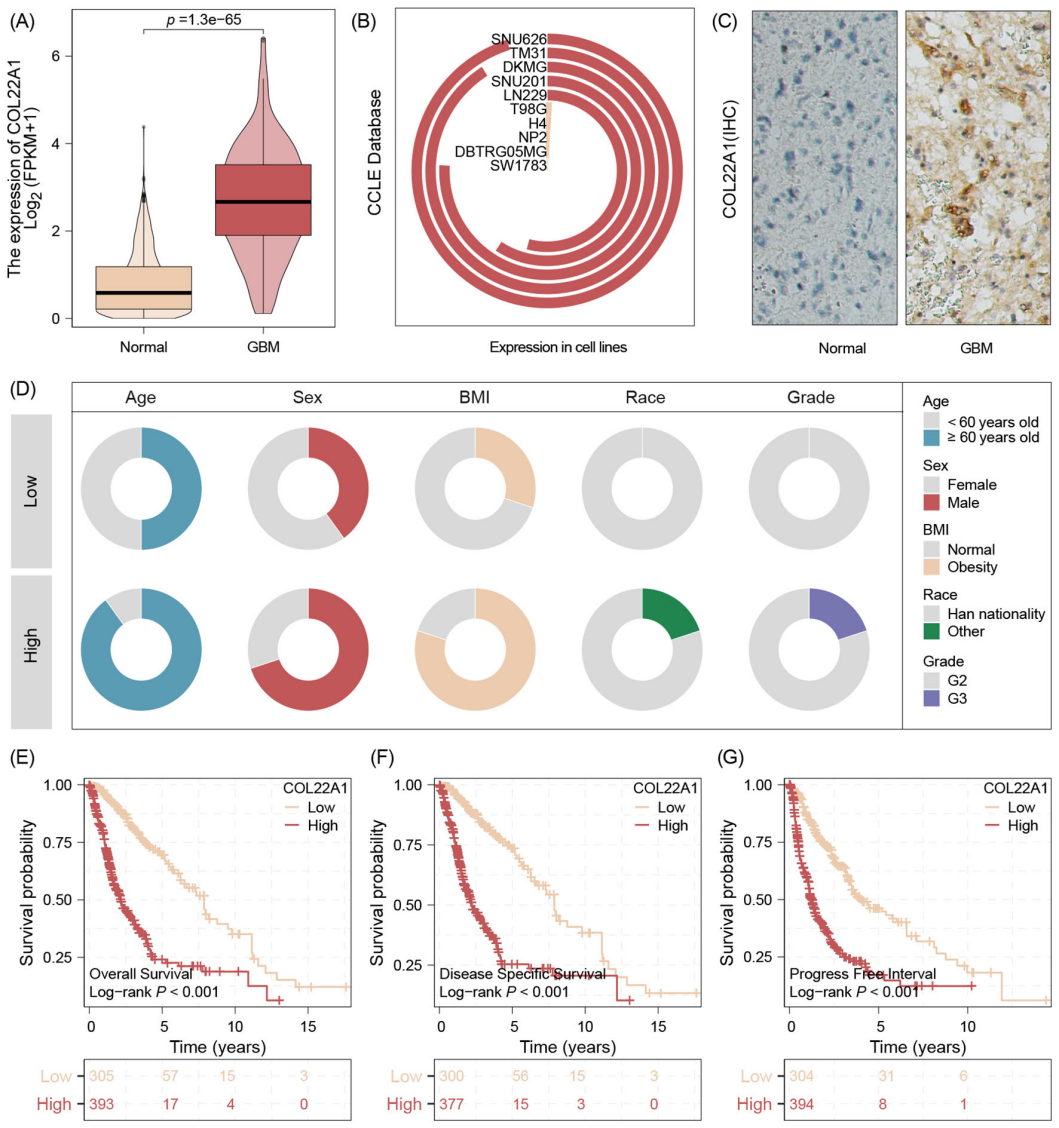
The transcriptome data analysis results validation that COL22A1 was high expression in GBM tissues while compared with normal tissues **(A)**. And the Cell lines results show that this gene expression high in SNU626 cell lines and low expression in SW1783 cell line **(B)**. The IHC results also demonstrate that this gene high expression in GBM tissues **(C)**. Patients with high expression had greater age and were more male, more obese patients, as well as high WHO grade **(D)**. The survival analysis show that high expression of COL22A1 was negative with survival rate, no matter OS,DFS or PFI **(E-G)**.

The transcriptome data analysis results validation that COL22A1 was high expression in GBM tissues while compared with normal tissues (**Figure 2A**). And the Cell lines results show that this gene expression high in SNU626 cell lines and low expression in SW1783 cell line (**Figure 2B**). Our center IHC results also demonstrate that this gene high expression in GBM tissues(**Figure 2C**). We further analyzed samples with high and low tumors suggested by immunohistochemical results. The results suggested that patients with high expression had greater age and were more male, more obese patients, as well as high WHO grade (**Figure 2D**). In addition, the survival analysis show that high expression of COL22A1 was negative with survival rate, no matter OS,DFS or PFI (**Figures 2E-G**).

### COL22A1 was associated with clinical factors

**Figure 3:**
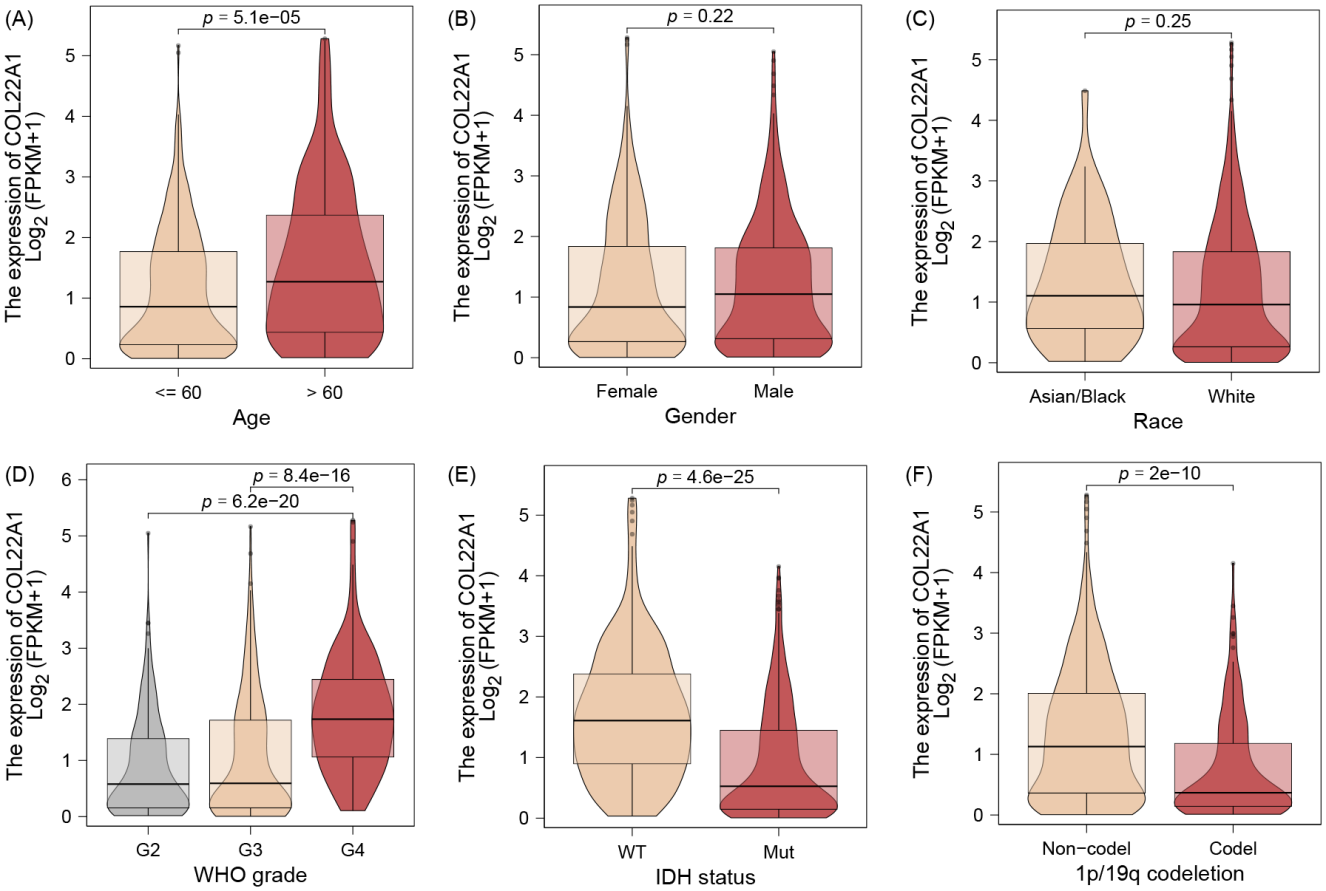
The results of the correlation analysis suggest that patients with high expression of the gene usually imply a higher age **(A)**. However, there was no difference in the expression of the gene by sex or race **(B, C)**. Interestingly, we observed a similar gradual increase in the expression of this gene as the WHO Grade increased **(D)**. In addition, we found that the expression of this gene was elevated in the WT and 1p/19q codeletion groups of IDH and was statistically different between the groups **(E, F)**.

The results of the correlation analysis suggest that patients with high expression of the gene usually imply a higher age (**Figure 3A**). However, there was no difference in the expression of the gene by sex or race (**Figures 3B, C**). Interestingly, we observed a similar gradual increase in the expression of this gene as the WHO Grade increased (**Figure 3D**). In addition, we found that the expression of this gene was elevated in the WT and 1p/19q codeletion groups of IDH and was statistically different between the groups (**Figures 3E, F**).

### COL22A1 was involved in multi-immune pathway and classical pathway

**Figure 4:**
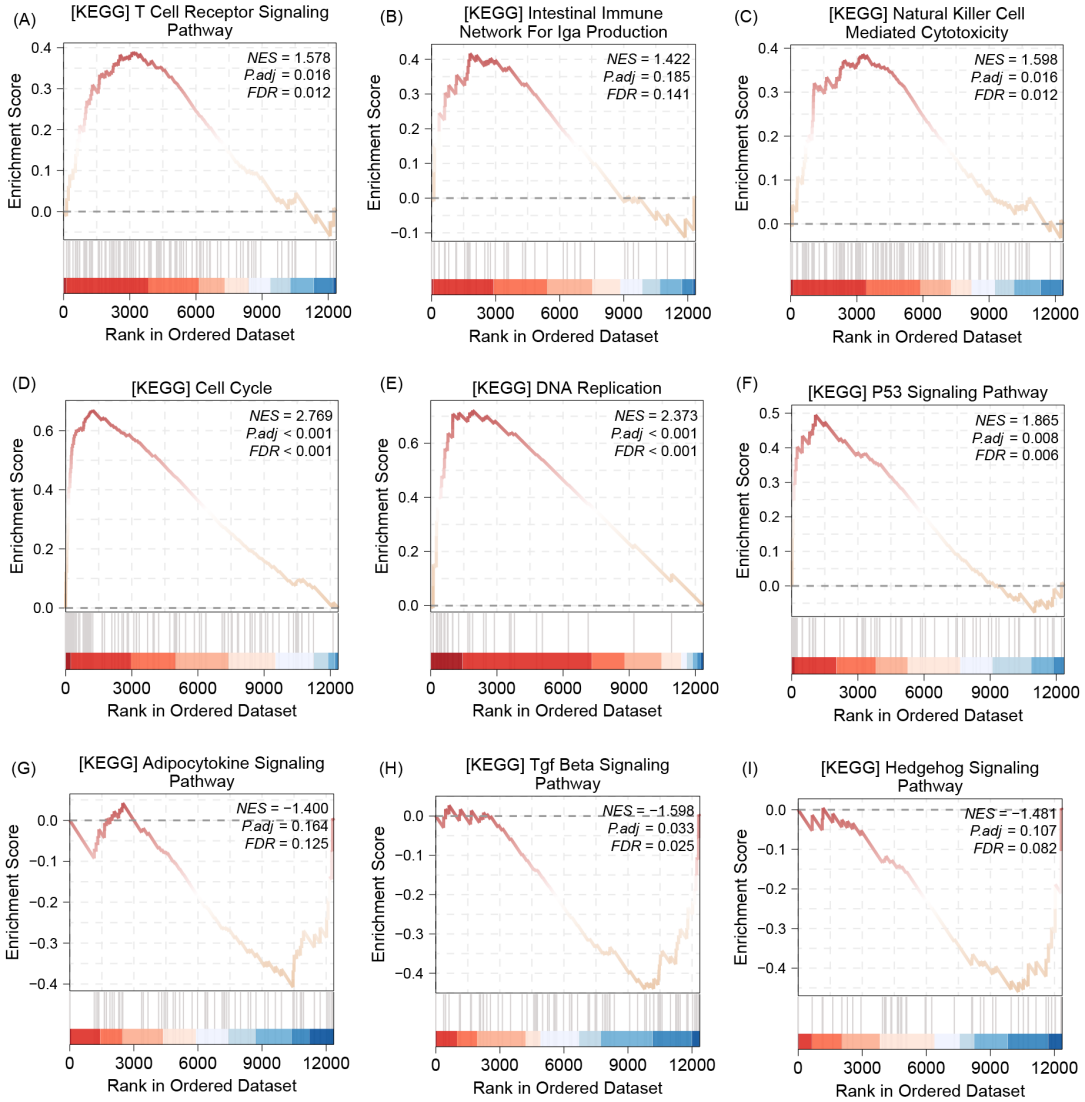
The GSEA analysis results show that, this gene could be enrichment in multi-immune pathway, such as T cell receptor signaling pathway **(A)**, intestinal immune network for lga production **(B)**, and Natural killer cell mediated cytotoxicity **(C)**. In addition, the enrichment results also demonstrate that COL22A1 was involved in cell cycle **(D)**, DNA replication **(E)**,P53 signaling pathway **(F)**, Adipocytokine signaling pathway **(G)**, Tgf Beta signaling pathway **(H)**, and hedgehog signaling pathway **(I)**.

The GSEA analysis results show that, this gene could be enrichment in multi-immune pathway, such as T cell receptor signaling pathway (**Figure 4A**), intestinal immune network for lga production (**Figure 4B**), and Natural killer cell mediated cytotoxicity (**Figure 4C**). In addition, the enrichment results also demonstrate that COL22A1 was involved in cell cycle (**Figure 4D**), DNA replication (**Figure 4E**), P53 signaling pathway (**Figure 4F**), Adipocytokine signaling pathway (**Figure 4G**), Tgf Beta signaling pathway (**Figure 4H**), and hedgehog signaling pathway (**Figure 4I**).

### COL22A1 was related to immune infiltration

**Figure 5:**
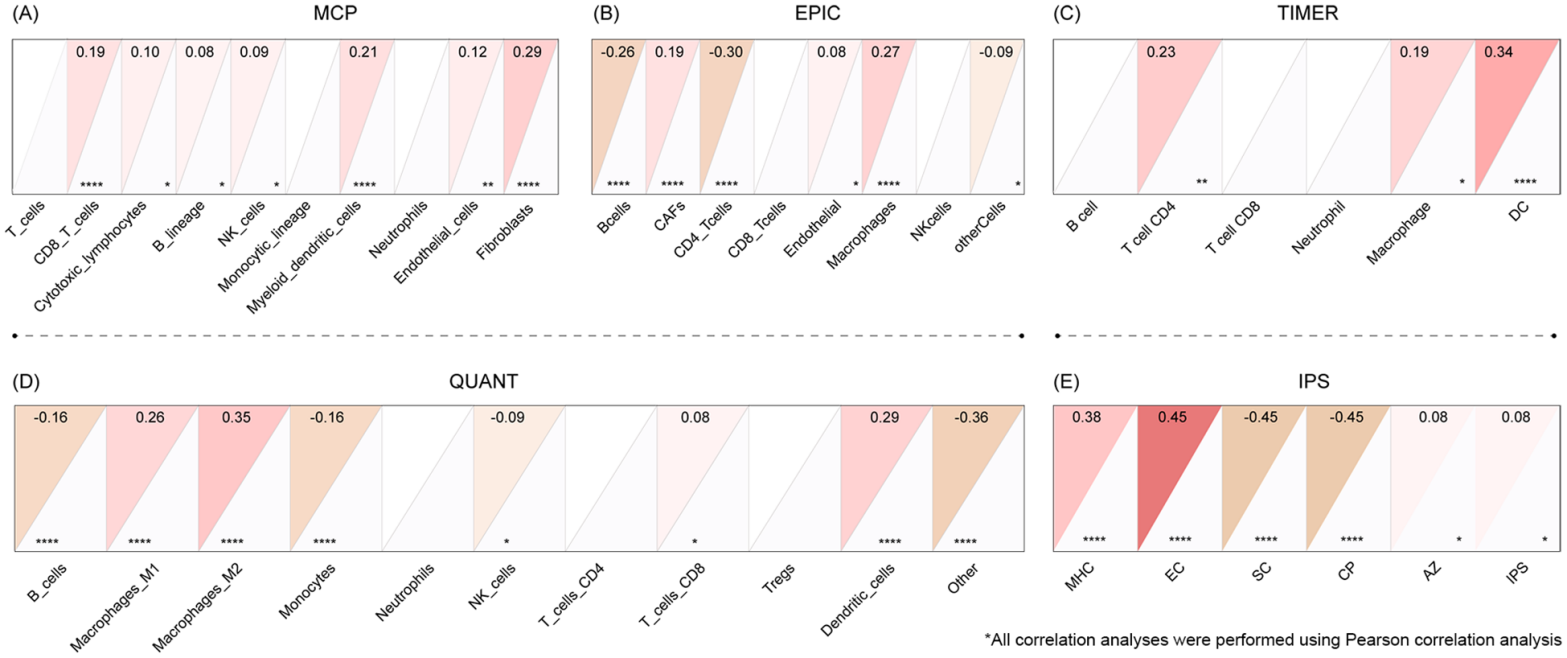
The expression of COL22A1 was found to be associated with immune infiltration across multiple analytical platforms. In the MCP-counter analysis, COL22A1 expression showed a positive correlation with CD8^+^ T cells, endothelial cells, and fibroblasts **(A)**. Similarly, the EPIC analysis revealed a negative correlation with B cells and CD4^+^ T cells, while showing a positive correlation with cancer-associated fibroblasts (CAFs) and macrophages **(B)**. TIMER analysis further confirmed a positive correlation between COL22A1 expression and macrophages as well as dendritic cells (DCs) **(C)**. In the QUANT analysis, COL22A1 expression was negatively correlated with B cells and positively correlated with M2 macrophages **(D)**. Finally, IPS analysis demonstrated a positive correlation with MHC molecules and endothelial cells (ECs), and a negative correlation with suppressive cells (SCs) and tumor-associated angiogenic factors (CPs) **(E)**.

The expression of COL22A1 was found to be associated with immune infiltration across multiple analytical platforms. In the MCP-counter analysis, COL22A1 expression showed a positive correlation with CD8^+^ T cells, endothelial cells, and fibroblasts (**Figure 5A**). Similarly, the EPIC analysis revealed a negative correlation with B cells and CD4^+^ T cells, while showing a positive correlation with cancer-associated fibroblasts (CAFs) and macrophages (**Figure 5B**). TIMER analysis further confirmed a positive correlation between COL22A1 expression and macrophages as well as dendritic cells (DCs) (**Figure 5C**). In the QUANT analysis, COL22A1 expression was negatively correlated with B cells and positively correlated with M2 macrophages (**Figure 5D**). Finally, IPS analysis demonstrated a positive correlation with MHC molecules and endothelial cells (ECs), and a negative correlation with suppressive cells (SCs) and tumor-associated angiogenic factors (CPs) (**Figure 5E**). These findings collectively suggest that COL22A1 plays a significant role in modulating the tumor immune microenvironment by interacting with diverse immune and stromal cell populations.

### COL22A1 was associated with immune checkpoints and immune regulated genes

**Figure 6:**
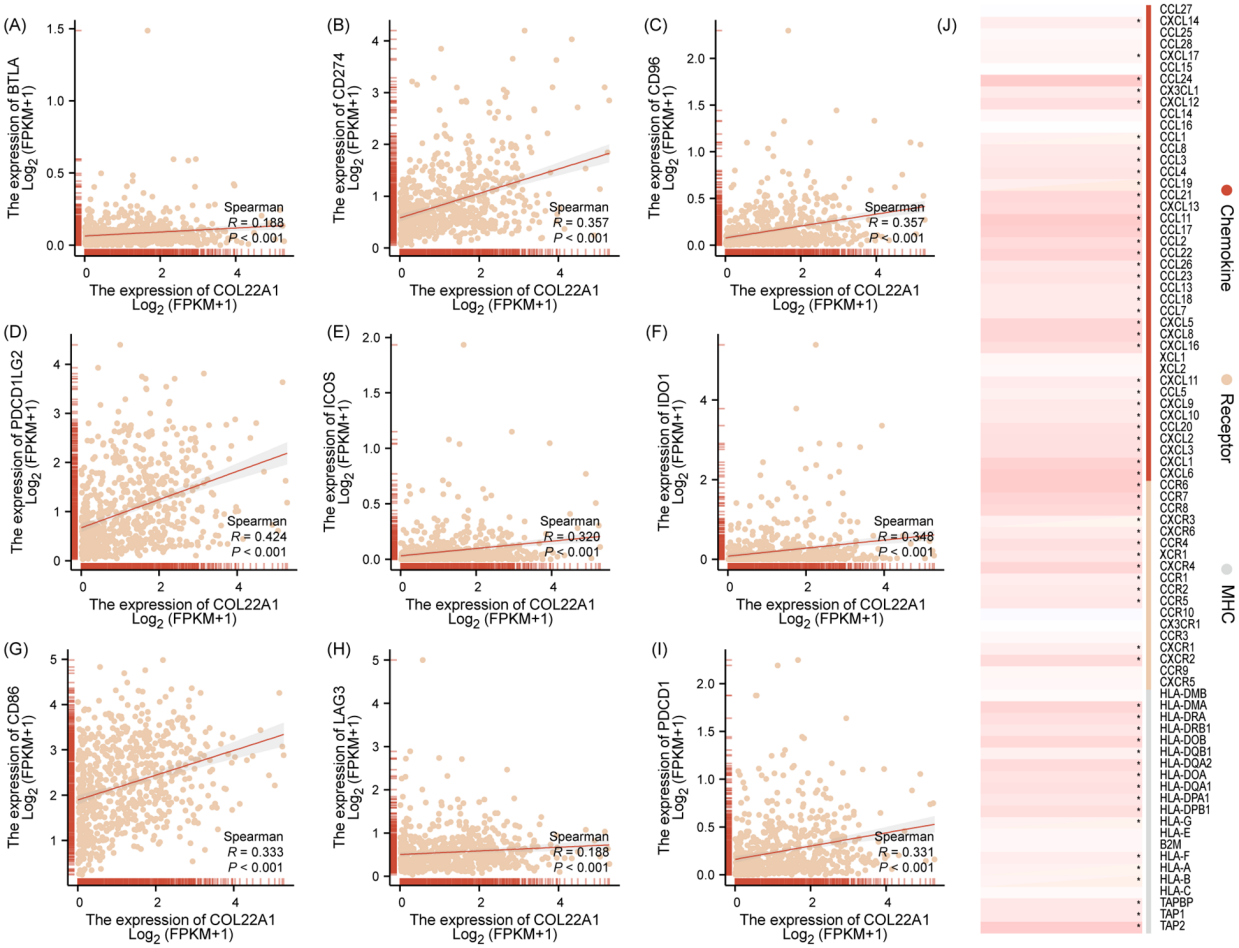
COL22A1 showed significant positive correlations with immune checkpoint molecules, including BTLA, CD274, CD96, PDCD1LG2, ICOS, IDO1, CD86, LAG3, and PDCD1 **(A–I)**. Additionally, COL22A1 exhibited correlations with immune-regulated genes, further supporting its role in modulating the immune microenvironment **(J)**.

The expression of COL22A1 was positively correlated with several immune checkpoints and immune-regulated genes, suggesting its involvement in immune modulation. Specifically, COL22A1 showed significant positive correlations with immune checkpoint molecules, including BTLA, CD274, CD96, PDCD1LG2, ICOS, IDO1, CD86, LAG3, and PDCD1 (**Figures 6A-I**). Additionally, COL22A1 exhibited correlations with immune-regulated genes, further supporting its role in modulating the immune microenvironment. These findings highlight the potential importance of COL22A1 in immune escape mechanisms and its relevance as a candidate for therapeutic targeting in immuno-oncology (**Figure 6J**).

## Discussion

In recent years, the application of multi-omics has revolutionized the diagnosis and prognostic prediction of cancers, offering unprecedented insights into complex biological systems and their interactions in various oncological conditions. Correspondingly, an increasing number of studies are applying multi-omic approaches to improve the diagnosis and prognostic stratification of GBM, facilitating more precise and targeted therapies (20). Yan et al (21). developed and validated a deep-learning imaging signature that significantly predicts survival outcomes in GBM patients, correlates with key biological pathways and genetic alterations, and outperforms traditional clinical molecular approaches in risk stratification, demonstrating an improved C index and net reclassification improvement. In addition, a previous study, by integrating clinical, radiomic, and genetic data, significantly enhanced the accuracy of predicting OS in IDH-wildtype GBM patients, with the combined omics approach improving the concordance index and achieving AUCs of 0.78 in the discovery cohort and 0.75 in the replication cohort (22). As in our study, we have integrated radiomics and genomics to identify five genes (*VAMP8*, *CYBA*, *COL22A1*, *FCER1G*, and *NPC2*) positively correlated with a risk score designed to predict the aggressiveness and potential outcomes of GBM, as well as its potential to trigger AE. This approach enhances our understanding of GBM’s complex behavior and its implications for AE, providing a more robust predictive model for clinical outcomes. Among these genes, *COL22A1* was identified as the hub gene, indicating its critical influence on the regulatory networks affecting GBM behavior and patient prognosis, as well as its potential role in driving AE. This highlights *COL22A1*’s importance not only in GBM progression but also in its potential to activate immune mechanisms involved in AE.

Collagen type XXII alpha 1 chain (*COL22A1*), a member of the collagen family, is known for its role in the structural matrix of connective tissues (23). Like many in its family, *COL22A1* and related collagen products are implicated in promoting tumor development, indicating its potential significance in cancer biology and tumor progression (24). In a recent study, *COL22A1* was identified as part of a group of extracellular matrix molecules that show elevated expression in the TME of GBM, suggesting its involvement in regulating the complex angiogenic processes within GBM and potentially impacting patient survival outcomes (25). In addition, *COL22A1* was found to be overexpressed and to play a pro-oncogenic role in GBM, as demonstrated by impaired proliferation, migration, and invasion of glioma cells following *COL22A1* silencing, underscoring its significance in tumor progression and as a biomarker for predicting patient survival (26). In our study, *COL22A1* was also shown to be highly expressed in multiple GBM cell lines and verified at the protein level. Furthermore, the expression of *COL22A1* is associated with common clinical risk factors such as older age, male gender, and higher tumor grade. Additionally, high expression of *COL22A1* correlates with poorer patient prognosis. These findings confirm the potential role of *COL22A1* in the progression of GBM and highlight its significance as a marker for prognosis and a target for therapeutic intervention. However, current research does not establish a direct link between the gene *COL22A1* and AE nor its impact on the immune profile of GBM, highlighting an area for future studies to explore potential connections that could provide deeper insights into the genetic and immunological underpinnings of these conditions.

Immunotherapy has dramatically altered the landscape of cancer treatment, with significant successes using checkpoint inhibitors and CAR T cells for various cancers (27), while GBM has consistently demonstrated robust resistance due to its unique intrinsic and adaptive immune evasion mechanisms (14, 28). For example, the CheckMate 143 trial, the first large randomized study targeting programmed death-1 (PD-1) signaling in GBM, demonstrated that nivolumab did not extend OS in patients with recurrent GBM compared to bevacizumab (29). The TME of GBM may partly explain the suboptimal outcomes observed, as a study has shown that the clinical response to anti-PD-1 immunotherapy in GBM is linked to specific genetic alterations, immune-related expression profiles, and the degree of immune cell infiltration, which collectively reflect the tumor’s clonal evolution and adaptive immune landscape during treatment (22, 30). Notably, we found that the expression of *COL22A1* positively correlates with common immune checkpoints such as PD-1 (*PDCD1*), PD-L1 (*CD274*), and LAG3 in GBM, suggesting that *COL22A1* may play a significant role in regulating immune evasion mechanisms in GBM. Additionally, DEGs between groups with high and low *COL22A1* expression were enriched in immune-related pathways such as the T Cell Receptor Signaling and Natural Killer Cell-Mediated Cytotoxicity pathways, suggesting a significant role for *COL22A1* in modulating immune responses in the TME. Moreover, a previous study has revealed that changes in the extracellular matrix (ECM) components, such as the loss of Hyaluronan and Proteoglycan Link Protein 1 (HAPLN1), significantly impacted immune responses and cell migration within the TME in aging skin (24). Given that *COL22A1* is known for its role in ECM organization and has been associated with tumor progression in other studies (31, 32), It is plausible that *COL22A1* could similarly influence the structural and functional dynamics of the ECM, potentially affecting interactions between immune cells and cancer cells. However, establishing a definitive link between *COL22A1* and the immune response requires further exploration.

Overall, we utilized machine learning to analyze radiomics data, constructing a risk model integrated with genomic data that identifies *COL22A1* as a hub gene. This model is designed not only to diagnose and predict the prognosis of GBM but also to assess the potential for GBM to drive AE. Building on this foundation, we further explored the relationship between *COL22A1* and the TME, discovering correlations with multiple immune cell checkpoints, chemokines, and MHC loci that influence immune responses, enriched in immune-related pathways. These findings highlight *COL22A1*’s potential as a therapeutic target and lay the groundwork for developing new immunotherapeutic strategies that could address both GBM prognosis and the likelihood of associated AE. However, our study has several limitations. Firstly, the impact of *COL22A1* on the survival of GBM patients and their response to immunotherapy has not been validated with real-world data. Secondly, the interaction between the tumor and its microenvironment is a complex process, and we did not explore the effects of *COL22A1* on the GBM and AE TME at the single-cell level. Additionally, although our research shows that *COL22A1* expression correlates with common immune checkpoints and MHC loci, suggesting its potential as a target for immune evasion, it remains unclear whether this effect is mediated through immune checkpoints or influences the interactions between immune and tumor cells via the extracellular matrix. Furthermore, the specific immune mechanisms through which GBM drives AE via *COL22A1* are still unclear. Further studies are needed to clarify these mechanisms.

## Data Availability

The datasets presented in this study can be found in online repositories. The names of the repository/repositories and accession number(s) can be found in the article/**Supplementary Material**.

## Supplementary material

Supplementary Table 1 Random Forest results of Top 5 features.

Supplementary Table 2 Lasso regression results.
