# Supplementary material for: Artificial intelligence-based radiogenomics reveals the potential immunoregulatory role of COL22A1 in glioma and its induced autoimmune encephalitis
Source: Front Immunol. 2025 Mar 6;16:1562070. doi: 10.3389/fimmu.2025.1562070 (PMC11922723; doi:10.3389/fimmu.2025.1562070)
Supplement: Supplementary Table 1 — Random Forest results of Top 5 features. [file Table1.docx]

| **Supplement Table 1 Random Forest results of Top 5 features** | | |
| --- | --- | --- |
| **ID** | **Importance** | **Radiomics Features** |
| F1 | 3.000449 | original_shape_Sphericity |
| F2 | 2.495783 | wavelet.LHL_firstorder_RootMeanSquared |
| F3 | 2.342078 | wavelet.HHH_firstorder_Mean |
| F4 | 2.255918 | wavelet.LHL_glrlm_LongRunLowGrayLevelEmphasis |
| F5 | 2.216549 | gradient_gldm_LargeDependenceHighGrayLevelEmphasis |
