# Supplementary material for: Artificial intelligence-based radiogenomics reveals the potential immunoregulatory role of COL22A1 in glioma and its induced autoimmune encephalitis
Source: Front Immunol. 2025 Mar 6;16:1562070. doi: 10.3389/fimmu.2025.1562070 (PMC11922723; doi:10.3389/fimmu.2025.1562070)
Supplement: Supplementary Table 2 — Lasso regression results. [file Table2.docx]

| **Supplement Table 2 Lasso regression results** | | |
| --- | --- | --- |
| **ID** | **Value** | **Radiomics Features** |
| F6 | 2.330810289 | original_shape_Sphericity |
| F7 | 0.981074812 | wavelet.HLL_firstorder_Mean |
| F8 | 1.089272447 | wavelet.HLH_firstorder_Mean |
| F9 | 1.023715205 | gradient_glszm_GrayLevelVariance |
